# Flare of Antiphospholipid Syndrome in the Course of COVID-19

**DOI:** 10.1055/s-0040-1716735

**Published:** 2020-09-11

**Authors:** Alexandre Thibault Jacques Maria, Isabelle Diaz-Cau, Jean-Marc Benejean, Anaïs Nutz, Aurélie Schiffmann, Christine Biron-Andreani, Philippe Guilpain

**Affiliations:** 1 Department of Internal Medicine–Multi-organ Diseases, Local Referral Center for Auto-immune Diseases, Montpellier School of Medicine, Saint-Eloi University Hospital, University of Montpellier, Montpellier, France; 2 IRMB, Inserm U1183, CHU Montpellier (Saint-Eloi University Hospital), University of Montpellier, Montpellier, France; 3 Hemostasis Laboratory, Referral Center for Hemophilia, Montpellier University Hospital, Saint-Eloi University Hospital, Montpellier, France; 4 Department of Radiology, Narbonne Hospital, Narbonne, France; 5 Department of Medicine and Endocrinology, Narbonne Hospital, Narbonne, France

**Keywords:** COVID-19, infection, thrombosis, antiphospholipid syndrome, hydroxychloroquine

## Abstract

We report the case of a 48-year-old man followed since 2013 for primary antiphospholipid syndrome (APLS) revealed by venous thromboembolism in the presence of antiphospholipid antibodies (APL-Abs, anticardiolipin and anti-β-2-glycoprotein-1), who decompensated in the course of coronavirus disease (COVID-19). Despite efficient anticoagulation, he suffered bilateral adrenal glands hemorrhage and limb arterial ischemia. The tropism of severe acute respiratory syndrome coronavirus-2 for endothelium can lead to microangiopathy and increased risk for thrombosis. If APL-Abs positivity can be an epiphenomenon under inflammatory and prothrombotic conditions, COVID-19 was herein responsible for disseminated thrombosis and a threat of catastrophic APLS, despite efficient anticoagulation.

Antiphospholipid syndrome (APLS) is an autoimmune systemic disorder characterized by thrombosis (involving arteries, veins, and/or small vessels) and/or obstetrical events (such as recurrent early pregnancy loss, fetal loss, or pregnancy morbidity) in association with persistent antiphospholipid antibodies (APL-Abs). 1 The new emerging coronavirus called “severe acute respiratory syndrome coronavirus-2” (SARS-CoV-2), responsible for the related disease COVID-19 has been reported as a de novo coagulopathy in the setting of APL-Abs. 2 Herein, we report a flare of APLS following COVID-19, suggesting that SARS-CoV-2 may also trigger thrombosis in pre-existing conditions.

A 48-year-old man treated since 2013 with vitamin K antagonists (VKA) for primary APLS revealed by venous thromboembolic event (VTE) in the presence of lupus anticoagulant (LA) and persistent APL-Abs (anticardiolipin [aCL] and anti-β2-glycoprotein-1 [aβ2GPI], immunoglobulin [Ig]M and IgG >40 UI/L). The medical history included nongenetic iron overload managed by venesection therapy between 2011 and 2018.

On March 20, 2020, he declared fever, cough, and myalgia, leading to hospitalization on March 25 (day 5). Nasopharyngeal swab was positive for SARS-CoV-2 and computed tomography scan showed typical diffuse lung involvement, without respiratory distress ( Fig. 1A ). Biology disclosed mild lymphopenia (0.75 G/L), an elevation of C-reactive protein (112 mg/L), the platelet count was normal (171 G/L), and INR 2.47 under fluindione. Arterial blood gas tests on ambient air showed mild hypoxemia (pO _2_ 75 mm Hg), and normal oxygen saturation (96%).

**Fig. 1:**
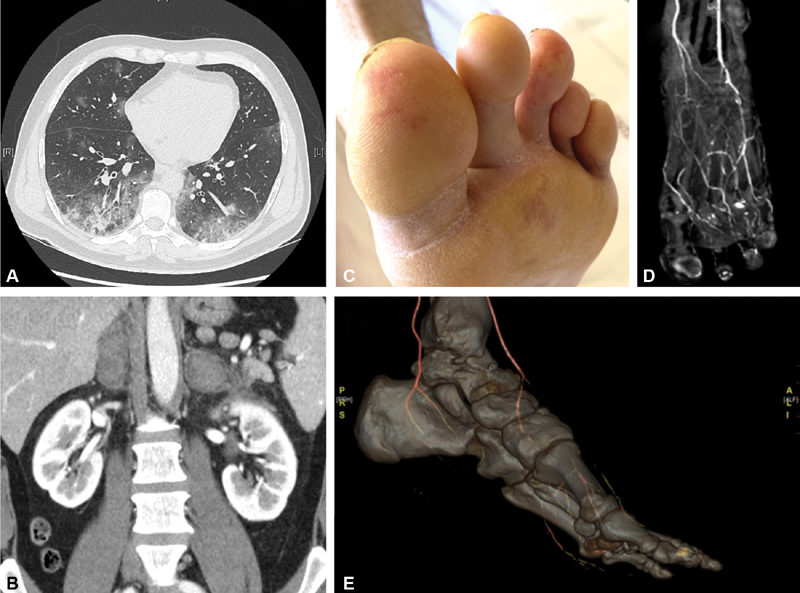
( **A** ) CT scan disclosing ground glass opacities and condensations consistent with a pattern of severe COVID-19 pneumonia; ( **B** ) CT scan disclosing bilateral adrenal gland hemorrhage; ( **C** ) left toes showing acral ischemic lesions; ( **D, E** ) angio-CT scan disclosing dorsalis pedis artery occlusion with 3D reconstruction.

He received hydroxychloroquine (400 mg a day for 7 days) and azithromycin (500 mg on day 1 followed by 250 mg for 4 more days) together with low flow oxygen for a couple of days. On day 12, while fever and pulmonary involvement had improved, the patient suffered sudden abdominal pain revealing bilateral adrenal glands hemorrhage ( Fig. 1B ). INR was still correct (2.7), but we observed high levels of fibrinogen (7 g/L) and D-dimers >2N (1,309 ng/mL), and decreased antithrombin III activity (63%). It was then decided to stop VKA and switch for low-molecular-weight heparin (enoxaparin 100 UI/kg twice a day). On day 17, painful acral ischemic lesions concerning left toes revealed dorsalis pedis artery occlusion ( Fig. 1C–E ). No VTE was found in association, embolic causes were ruled out, and positivity for LA and APL-Abs was confirmed. Treatment was switched for intravenous continuous unfractionated heparin (anti-Xa target 0.5–0.7) and no other clinical or radiological thrombotic event occurred thereafter, excluding catastrophic APLS. The patient was eventually discharged on April 16 (day 27) under VKA and substitutive opotherapy for adrenal function.

In addition to an increased risk for adverse outcome in patients with prior cardiovascular disease, COVID-19 can also lead to an hypercoagulability state 3 resulting in vascular and thrombotic events, such as ST-segment elevation coronary syndromes, 4 pulmonary embolism, 5 and disseminated intravascular coagulation. 6 In this context, Tang et al 7 reported the prognosis value of abnormal coagulation parameters (high D-dimer and fibrin degradation product levels). All these findings prompted the International Society on Thrombosis and Haemostasis to edit recommendations for anticoagulant therapy in COVID-19. 8 The underlying cardiovascular risk factors, critical illness with hypoxemic conditions, hemostatic factors, and intense inflammatory response were considered initially to predispose to vascular events in COVID-19. Whether thrombosis is linked to some mechanisms specific to SARS-CoV-2 is now considered as a possibility but is still to be fully demonstrated. Interestingly, microthrombi have been found within the lungs of COVID-19 patients according to autopsy studies 9 and also within skin lesions. 10 A thrombotic microangiopathy might contribute to COVID-19 injury in some cases, in association with the activation of the alternative and lectin pathways of complement system 11 12 13 and review. 14 The complement system is even being considered as a target for therapy in COVID-19. 15 16 17

In April, Zhang et al 2 reported multiple cerebral infarctions in three COVID-19 patients with APL-Abs (aCL IgA, aβ2GPI IgA and IgG). This presentation was suggestive of APLS but did not meet the criteria in terms of antibody isotype, titer, and persistence. 18 Notably, APL-Abs (mostly aCL antibodies) may be detected in the course of viral infections and/or critical illness. In these settings, they may be the markers of endothelial damage and may inconstantly be associated with thrombotic events. 19 Similarly, LA has been reported as a frequent feature of COVID-19, but ongoing heparin treatment may interfere with the LA detection and false-positive tests cannot be excluded. 20 In this study, among 56 COVID-19 patients, 45% were positive for LA and only 10% positive for aCL or aβ2GPI. Since then, other reports have discussed the pathogenic role of LA and/or APL detected in COVID-19 patients, considering that APL could be transient and were not always associated with thrombotic manifestations. 21 22 23 However, some patients with higher and persistent levels of APL or multiple APL positivity may be more at risk of cerebral infarction during COVID-19. 24

On the whole, in some patients, APL-Abs may be present prior to COVID-19, as a latent autoimmune condition that could be precipitated by SARS-CoV-2, as described in catastrophic APLS. 1

Furthermore, in a model of in vitro engineered human blood vessel organoids, SARS-CoV-2 shows a special tropism for endothelium through the expression of its receptor angiotensin converting enzyme 2. 25 In addition, lesions of endotheliitis appear to be induced by SARS-CoV-2, as demonstrated by the presence of viral particles within endothelial cells, together with inflammatory cells, resulting in apoptosis of these cells. 26 Thus, as suggested in our observation, SARS-CoV-2 may favor the pathogenic effects of APL (including the activation of endothelial cells, monocytes, platelets, and complement), resulting in proinflammatory/prothrombotic states. In a broader view, the endothelial tropism of SARS-CoV-2 may also modify the clinical presentation of COVID-19 in susceptible patients and it might be hypothesized that it triggers flares of underlying vascular diseases.

In the present case, we can also speculate that SARS-CoV-2 may have been responsible for thrombosis through a “two-hit hypothesis” in a patient with persistent APL. Moreover, we cannot exclude that the modification of anticoagulant therapy (i.e., switch for low-molecular-weight heparin) may also have contributed to APLS flare. However, bilateral adrenal glands hemorrhage (a rare but typical feature of APLS) was observed prior to the switch, which suggests that the thrombotic process was already ongoing. So, taken together, our findings and literature data suggest that COVID-19 represents a high-risk condition for APLS flare.

If APL-Abs positivity can be an epiphenomenon under inflammatory and prothrombotic conditions, COVID-19 was herein responsible for disseminated thrombosis and a threat of catastrophic APLS, despite efficient anticoagulation.
